# Inhibition of Human Dyskerin as a New Approach to Target Ribosome Biogenesis

**DOI:** 10.1371/journal.pone.0101971

**Published:** 2014-07-10

**Authors:** Laura Rocchi, Arménio J. M. Barbosa, Carmine Onofrillo, Alberto Del Rio, Lorenzo Montanaro

**Affiliations:** 1 Department of Experimental, Diagnostic and Specialty Medicine, Alma Mater Studiorum, University of Bologna, Bologna, Italy; 2 Surgical Pathology Unit, University Hospital of Parma, Parma, Italy; 3 “Giorgio Prodi” Interdepartmental Cancer Research Centre, Alma Mater Studiorum, University of Bologna, Bologna, Italy; 4 Institute for Organic Chemistry and Photoreactivity, National Research Council, Bologna, Italy; The Scripps Research Institute, United States of America

## Abstract

The product of the DKC1 gene, dyskerin, is required for both ribosome biogenesis and telomerase complex stabilization. Targeting these cellular processes has been explored for the development of drugs to selectively or preferentially kill cancer cells. Presently, intense research is conducted involving the identification of new biological targets whose modulation may simultaneously interfere with multiple cellular functions that are known to be hyper-activated by neoplastic transformations. Here, we report, for the first time, the computational identification of small molecules able to inhibit dyskerin catalytic activity. Different *in* *silico* techniques were applied to select compounds and analyze the binding modes and the interaction patterns of ligands in the human dyskerin catalytic site. We also describe a newly developed and optimized fast real-time PCR assay that was used to detect dyskerin pseudouridylation activity *in* *vitro.* The identification of new dyskerin inhibitors constitutes the first proof of principle that the pseudouridylation activity can be modulated by means of small molecule agents. Therefore, the presented results, obtained through the usage of computational tools and experimental validation, indicate an alternative therapeutic strategy to target ribosome biogenesis pathway.

## Introduction

One basic prerequisite for the development of antineoplastic therapeutics is represented by the identification of cellular processes that are selectively altered in cancer cells and that could be modulated by pharmacological actions on specific biological targets.

Among a series of cellular processes, both ribosome production and telomerase functions are known to be hyper-activated by neoplastic transformation. On one side, the rate of ribosome biogenesis regulates cellular growth and proliferation, and cancer cells carry over an increased production of ribosomes to sustain the protein synthesis necessary for unbridled cell growth [1], [2]. On the other hand, the reactivation of telomerase, allowing the maintenance of chromosome ends during cell proliferation, is a characteristic of about 85–90% of primary tumors. Although it is not detectable in most somatic cells, with the exception of some adult pluripotent stem cells, proliferative cells of renewal tissues, and male germline cells [3], [4]. Therefore the specific targeting of each of these two cellular processes has been explored for the development of drugs in order to selectively or preferentially kill cancer cells [5]–[8].

The product of DKC1 gene, dyskerin, is necessary for both processes of ribosome biogenesis and telomerase complex stabilization [9]. Indeed, dyskerin mediates the site specific uridine conversion to pseudouridine in rRNA and snRNA. Uridine modification in rRNA represents an early and crucial step of rRNA processing affecting the rate and the efficiency of ribosome production [10], [11]. In addition, dyskerin also binds the telomerase RNA component (TERC), stabilizing the telomerase enzymatic complex and the mutations of the DKC1 gene at specific sites or the reduction of its expression strongly reduces the levels of TERC and the activity of telomerase [9]. In the rare multisystemic syndrome X-linked dyskeratosis congenital and in a subset of human tumors arising in the general population, dyskerin has been proposed to act as a tumor suppressor [9], [10], [12]. In contrast, dyskerin is overexpressed in a number of human cancer types and high levels of dyskerin expression in tumors are associated with an aggressive clinical behavior in various tumor types including breast [10], prostate [11], head and neck [13], colon [14], and hepatocellular carcinomas [15]. These contrasting observations may be explained considering that in some cases the partial lack of dyskerin function could in the long term promote peculiar neoplastic features, while in a distinct subset of aggressive tumors the need to support the increased production of ribosomes and the increased demand for telomerase function characterizing actively growing tumor cells requires dyskerin overexpression.

This is consistent with the need to support the increased production of ribosomes and the increased demand for telomerase function characterizing actively growing tumor cells.

The targeting of dyskerin is therefore expected to weaken both the production of ribosomes and the proper telomerase complex functioning impairing preferentially the growth of highly proliferating cancer cells. Based on these facts we envisioned to specifically targeting dyskerin catalytic function by means of small molecule inhibitors in order to preferentially target cancer cells. To this end we have generated a structural model of the full-length human dyskerin based on known crystal structures of yeast [16] and screened, *in* *silico*, the Open National Cancer Institute database to select potential small molecule compounds interacting with the dyskerin catalytic site that was found particularly amenable for accommodating ligands [17], [18].

The effects of the selected compounds were tested with a newly developed and optimized fast real-time PCR assay detecting the dyskerin pseudouridylation activity *in* *vitro*. Among all compounds, we identified four agents capable of significantly inhibit dyskerin activity. This result represents the proof of principle indicating that dyskerin catalytic activity can be modulated pharmacologically by means of small molecule inhibitors.

## Results and Discussion

### Computer-aided modeling and molecular design

**Figure 1:**
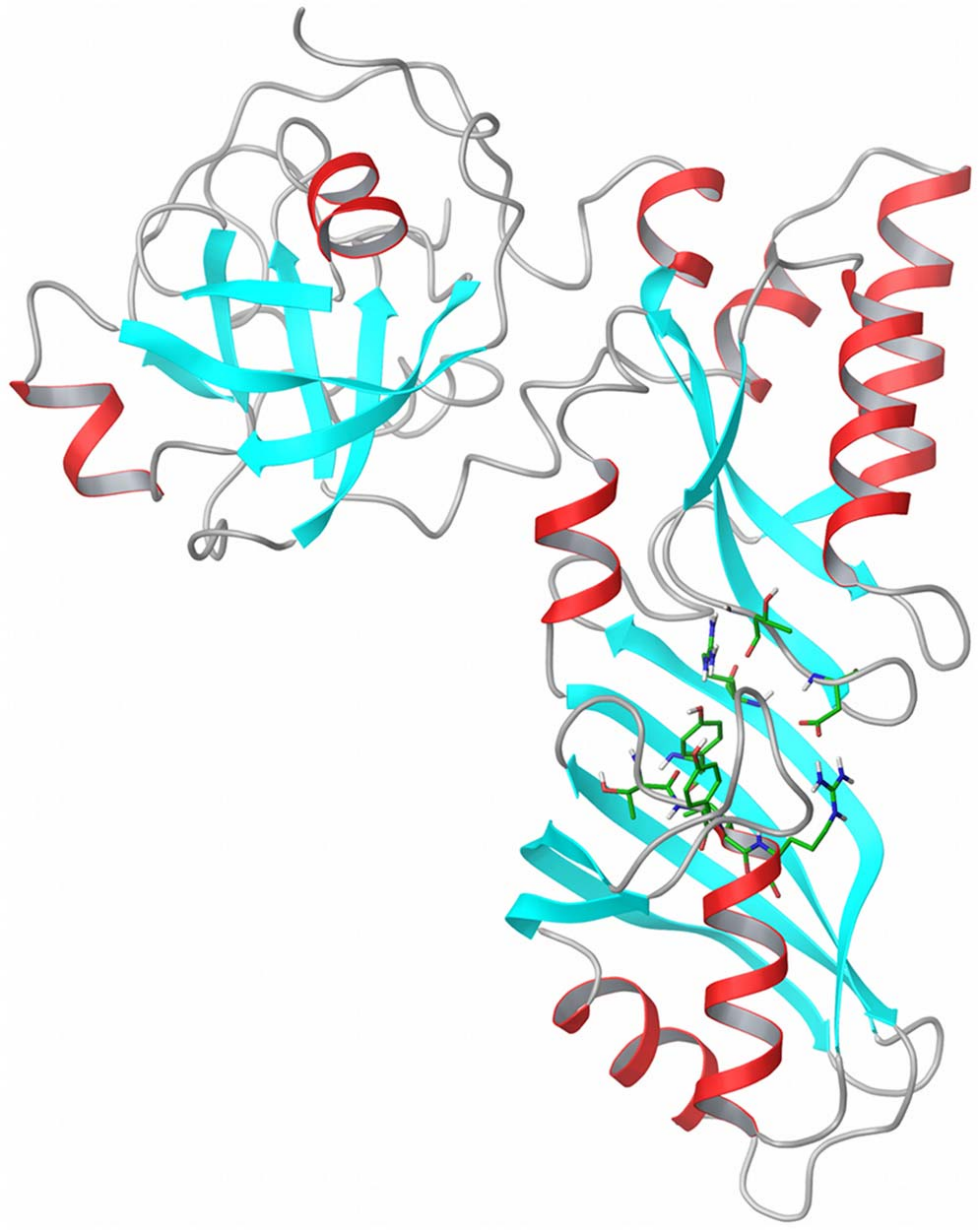
Structure of the homology model of human dyskerin. The model is based on the template of *saccharomyces cerevisiae* crystallographic structure of the Cbf5-Nop10-Gar1 complex (PDB id: 3U28). Important residues for pseudouridylation are highlighted to identify the catalytic region on the whole protein model. The present structure was obtained using MODELLER with the sequence alignment in Figure S1 in File S1. The Figure was produced with Maestro.

The three-dimensional structure of the human dyskerin structure has not been solved yet. In order to pursue a structure-based identification of dyskerin inhibitors, we created a homology model of human dyskerin by using a template crystal structure from *saccharomyces cerevisiae* (PDB ID: 3U28), which has a sequence identity of 73% with the human dyskerin sequence (Figure 1 and Figure S1 in File S1) [16]. The next more similar structure was found to be the prokaryotic cbf5 of *pyrococcus furiosus* (PDBID: 2EY4), which had a lower sequence alignment identity of 40% and therefore was not further considered [19]. The quality of the constructed model was assessed with the Procheck suite [20], and a molecular dynamics simulation that demonstrated the preservation of the model protein folding with a maximum backbone RMSD fluctuation of 2.5Å (Figure S2 in File S1). These results highlighted the good quality of the homology model obtained starting from the yeast structure (Figure 1).

Encouraged by this, and with the aim to identify new small molecule inhibitors targeting the human dyskerin catalytic site, we used the three-dimensional coordinates of the obtained homology model to set up a structure-based virtual screening approach. By means of molecular docking techniques, we screened *in* *silico* the open NCI compounds dataset of the CoCoCo databases [18] and the first hundreds of hits were visually inspected against different criteria (see Materials and Methods). We selected 14 molecules (Figure S3 in File S1) that were acquired from the NCI for experimental testing.

### Effect of selected molecules on cellular pseudouridylation activity and viability

**Figure 2:**
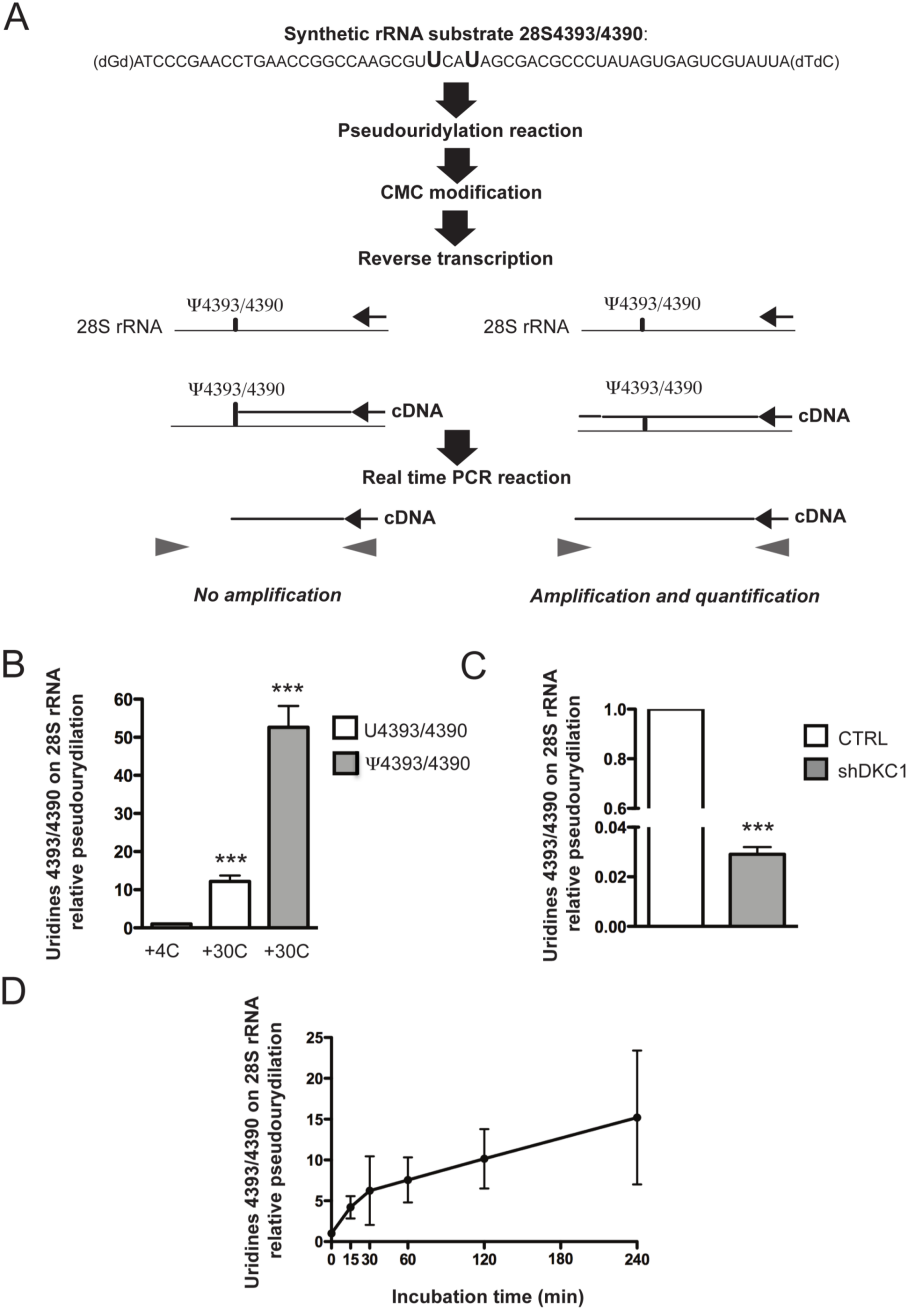
Pseudouridylation assay design and set up. (*A*) Schematic representation of the developed pseudouridylation real-time PCR-based assay. (*B*) Semi-quantitative evaluation of dyskerin pseudouridylation activity estimating the reverse-transcribed target products by real-time PCR. The same amount of two synthetic target RNAs, two different synthetic target RNA, one containing uridines in positions 4393 and 4390 and one containing pseudouridines in the same positions were subjected to nuclear lysate incubation, CMC modification, specific reverse transcription and real time PCR. Experiments were carried out at 4° and 30°C for 120 minutes. Histograms represent means and SDs from three independent experiments. *** = p<0.001). P value<0.05 is considered significant. (*C*) Semi-quantitative evaluation of dyskerin pseudouridylation activity in a nuclear lysates from control (CTRL) and dyskerin depleted cells (shDKC1). Experiments were carried out at 30°C for 60 minutes. Histograms represent means and SDs from three independent experiments. *** = p<0.001. P value<0.05 is considered significant. (*D*) Time course of the pseudouridylation reaction. The reactions were carried out at 30°C for 0, 15, 30, 60, 120, and 240 minutes and uridines U4393/U4390 were then verified by real-time PCR. Means and SDs from three independent experiments are represented.

To test the effect of the selected molecules on dyskerin pseudouridylation specific activity, we developed an *in* *vitro* real-time PCR-based assay (Figure 2A). A synthetic rRNA was used as a substrate and contained a small amount of the 28S rRNA sequence with uridines U4393/U4390, which are normally converted into a pseudouridine by dyskerin, and a 20 nucleotides long sequence at the 3′ to specifically reverse-transcribe the rRNA substrate. The synthetic rRNA substrate was incubated with a nuclear cellular lysate to produce the pseudouridylated RNA product. To detect substrate modification, the RNA product was chemically modified after lysate incubation using the [N-cyclohexyl-N’-β-(4-methyl-morpholinium)ethylcarbodiimide] (CMC) in order to generate N3-CMC-ψ adducts. While CMC is known to bind U and G bases, these adducts are readily cleaved by weakly alkaline conditions. Conversely, cleavage of N3-CMC-ψ requires strong alkaline conditions, and a combination of reactions with CMC and alkaline cleavage can be made specific to CMC addition to ψ. The obtained RNA product was subsequently reverse-transcribed specifically. The CMC-ψ complex efficiently blocks reverse transcription creating a truncated PCR product, which is not amplified through the successive real-time PCR (as experimentally demonstrated in Figure S4 in File S1). As a consequence, the assay allows a semi-quantitative evaluation of dyskerin pseudouridylation activity by estimating the reverse-transcribed target products by real-time PCR.

We first tested the efficiency of the in vitro assay (Figure 2B–D). In order to evaluate the capacity of our system to detect the presence of pseudouridines in selected positions, we tested our system using two different synthetic target RNA, one containing the uridines in positions 4393 and 4390 (U4393/4390) and one containing pseudouridines in the same positions (ψ4393/4390). To evaluate dyskerin pseudouridylation activity, both synthetic RNAs were incubated in a nuclear lysate at 30°C because this temperature represents the optimal condition for maximal enzymatic activity [21]. As an additional control we incubated the U4393/4390 synthetic rRNA substrate with the nuclear cellular lysate at 4°C, a condition in which the enzymatic reaction is down regulated. Our results showed that the rate of pseudouridylation of uridines U4393/U4390 at 30°C is significant increased (up to 12-fold) as respect to the same conditions at 4°C. On the other side, the comparison between the U4393/4390 and the ψ4393/4390 rRNAs allowed to demonstrate that a further increase in the level of pseudouridines can be measured by our system. (Figure 2B). Moreover, we evaluated the pseudouridylation activity by means of our assay in a nuclear lysate obtained from dyskerin depleted cells by DKC1 specific shRNA overexpression (shDKC1) and in their relevant control [22]. Lysates from dyskerin depleted cells displayed a reduced pseudouridylation activity with respect to the control lysate (Figure 2C). These controls demonstrate that dyskerin pseudouridylation activity is measured by the *in* *vitro* assay developed. We then evaluated pseudouridylation activity after 15, 30, 60, 120 and 240 minutes of the reaction incubation (Figure 2D). Results confirm that 120 minutes of incubation represent the best condition to appreciate the differences among the samples. To our knowledge, this is the first technology enabling the quantification of dyskerin catalytic activity without the use of radiolabelled tracers and the chromatographic separation of labeled nucleosides.

**Figure 3:**
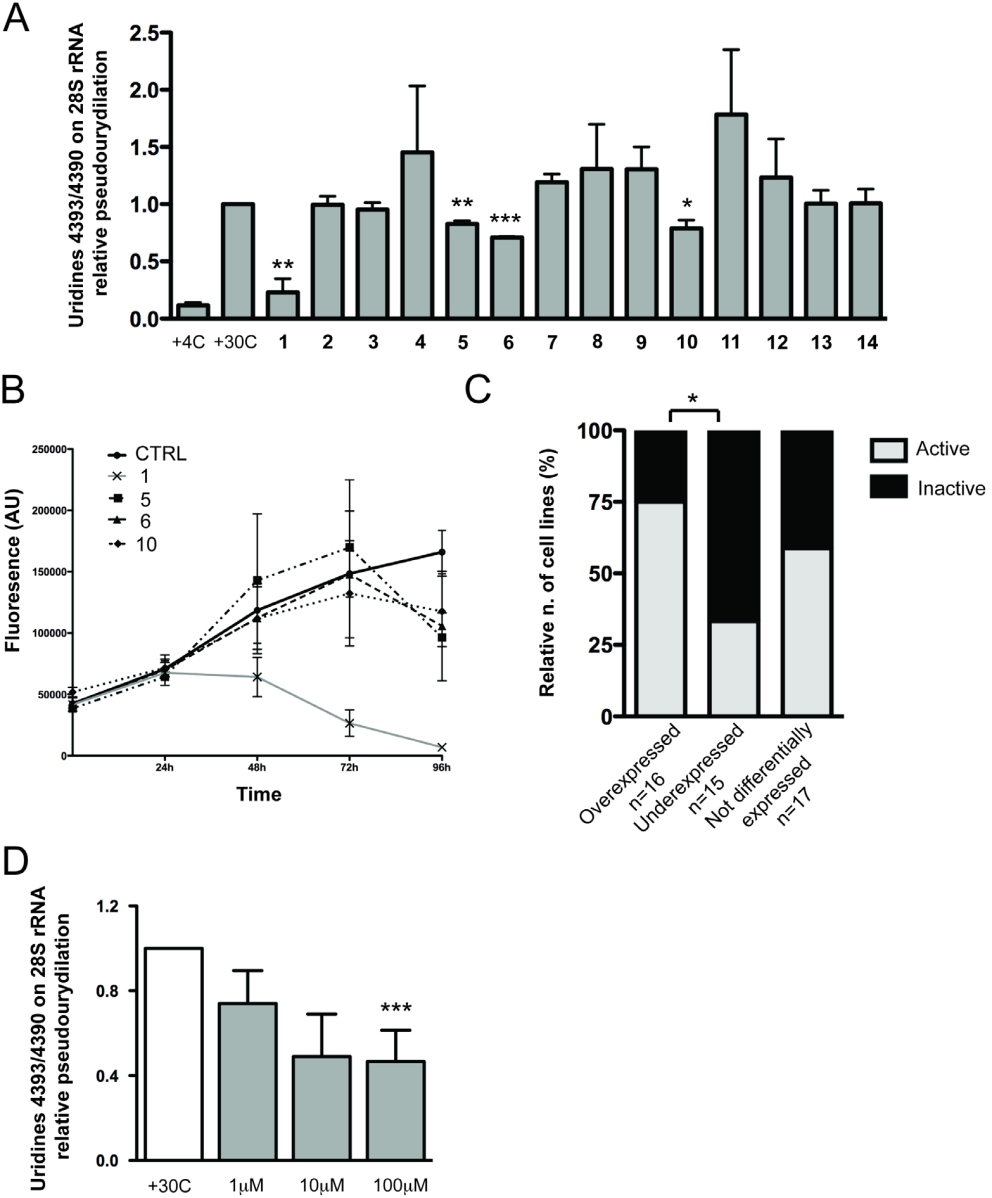
Effect of selected molecules on cellular pseudouridylation activity and viability. (*A*) Bioactivity of the 14 selected compounds against pseudouridylation activity of human dyskerin *in* *vitro*. All the reactions were carried out at 30°C for 120 minutes with 100 µM of each compounds or DMSO. Histograms represent means and SEMs from three independent experiments. P values<0.05 are considered significant and are indicated with *(p<0.05), **(p<0.01) and ***(p<0.001). (*B*) Cytotoxicity of compounds **1**, **5**, **6** and **10** on MCF7 cell line. 100 µM of compound **1**, **5**, **6** and **10** were added to cell and Alamar Blue assay performed each 24 hours for 4 days. Means and SDs from three independent experiments are represented. (*C*) Correlation between the expression profiles of DKC1 gene and reported bioactivity experiments of pyrazofurin (compound **1**). Cells lines are divided into three groups according to dyskerin expression: overexpressed, underexpressed and not differentially expressed. Histograms represent the percentage of the cell lines in each group sensitive to the pyrazofurin cytotoxic activity. * = p<0.05. P value<0.05 is considered significant. The P is not indicated where correlations between the groups are not significant. (*D*) Effect of treatment with compound **1** on MCF7 endogenous U4393/U4390 rRNA pseudouridylation. Cells were treated with 1 µM, 10 µM and 100 µM of compound **1** or DMSO for 24 hours. Histograms represent means and SEMs from three independent experiments. *** = p<0.001. P value<0.05 is considered significant.

We then tested the effect of selected molecules on dyskerin pseudouridylation activity using the assay described above (Figure 3A). In particular, we incubated each compound at 100 µM concentration in the reaction mixture for 120 minutes. The results showed that, among 14 tested compounds, compounds **1**, **5**, **6** and **10** significantly reduced the specific pseudouridylation activity on 28S uridines U4393/U4390, although to different extents (Table 1 and Figure S3 in File S1). This result indicates, for the first time, the possibility to target the dyskerin catalytic site with small molecules. As a consequence, it is possible to postulate the use of small molecule inhibitors as a new approach to target dyskerin catalytic activity in tumors with an increased dyskerin expression.

**Table 1:** Identity of chemical compounds (chemical structures available in Figure S3 in File S1) and their relative pseudouridylation measurements.

	Relative pseudouridylation (Mean ± SEM)
**1**	0.2304±0.1183
**5**	0.8283±0.02631
**6**	0.7101±0.0064
**10**	0.7879±0.07261

Compounds were tested at 100 µM concentration.

To test the biological effect of the four agents found, we evaluated their cytotoxicity using the Alamar Blue method. We performed the assay treating the MCF7 breast cancer cell line up to 96 hours. As shown in Figure 3B compounds **5**, **6**, and **10** had no cytotoxic effect in respect to control during the first 72 hours of treatment and the effect was detected only during the last 24 hours. On the contrary, compound **1** had a significant cytotoxic activity starting from 24 h of treatment. The toxicity of compound **1** was confirmed by the assessment of a dose/response curve (Figure S5 in File S1).

By targeting the catalytic activity of dyskerin, we intended to affect preferentially uridine modification and not other non-catalytic functions of the protein such as TERC stabilization [9]. The obtained results indicated that, in the employed experimental conditions, compound **1** has no significant effect on TERC levels (Figure S6 in File S1).

Moreover, we tested whether the effect of compound **1** on pseudouridylation activity is present not only on a nuclear lysate but on *in* *vitro* cultured cells. For this purpose we exposed MCF7 cells to different concentrations of compound **1** and, adapting our RT-real time PCR approach, we measured on the endogenous modification of the same uridine pair (28S rRNA U4393/U4390) tested in the in cell free assay. Obtained results indicate that endogenous 28S rRNA U4393/U4390 modification is inhibited by compound **1** (Figure 3C). Importantly, the treatment with a compound displaying no effect on dyskerin pseudouridylation activity in the cell free assay (compound **9**) did not induce any change on the modification of the same sites in cells (Figure S7 in File S1).

Compound **1** is also known as pyrazofurin, a known inhibitor of the orotodine-5′-monophosphate-decarboxylase (ODCase) involved in the *de* *novo* synthesis of pyrimidines nucleotides, DNA synthesis blocking and cellular replication [23], [24]. As a consequence, it is possible to hypothesize that the cytotoxic activity observed was driven by the inhibition of both dyskerin and ODCase. The use of this compound has been reported in several clinical trials for ovarian carcinoma [25], sarcoma [26], [27], colorectal carcinoma [28], acute myelogenous leukemia [29], breast cancer [30], lung cancer [31], melanoma [32] and others [33]. While these trials revealed a limited therapeutic potential of pyrazofurin, previous studies never considered dyskerin expression. Thus, it is not possible to exclude the possibility that this compound may be highly active on a selected group of patients with dyskerin overexpression. In order to understand whether possible patterns could arise between overexpression profile of human dyskerin and bioactivity data, we envisioned highlighting possible correlations from reported data of compounds **1**, **5**, **6** and **10** and the expression of DKC1 gene in human cancer cell lines (Table S1 and S2 in File S1). Only for compound **1**, pyrazofurin, we could retrieve a significant set of bioactivity data from the PubChem database. We found that, in human cancer cell lines overexpressing the DCK1 gene, there is a significant greater incidence of reported activity of pyrazofurin as respect to cell lines in which the DKC1 gene is underexpressed (p = 0.03) (Figure 3D). These results suggest that dyskerin expression may be an important factor in determining the activity of pyrazofurin.

### Compound structure and activity analysis

The results presented in Figure 3 reveal for the first time the possibility for small molecules to interact with dyskerin catalytic activity. Thus, the identification of chemical scaffolds able to inhibit dyskerin function constitutes the first step towards the design of *ad-hoc* molecules with improved enzymatic and biological effects. In respect to this, and with the purpose to rationalize at a molecular level the bioactivity of compounds **1**, **5**, **6** and **10**, we performed a thoughtful structural analysis of the molecules and a summary of the interactions in the catalytic site that are responsible to ligand - dyskerin binding.

**Figure 4:**
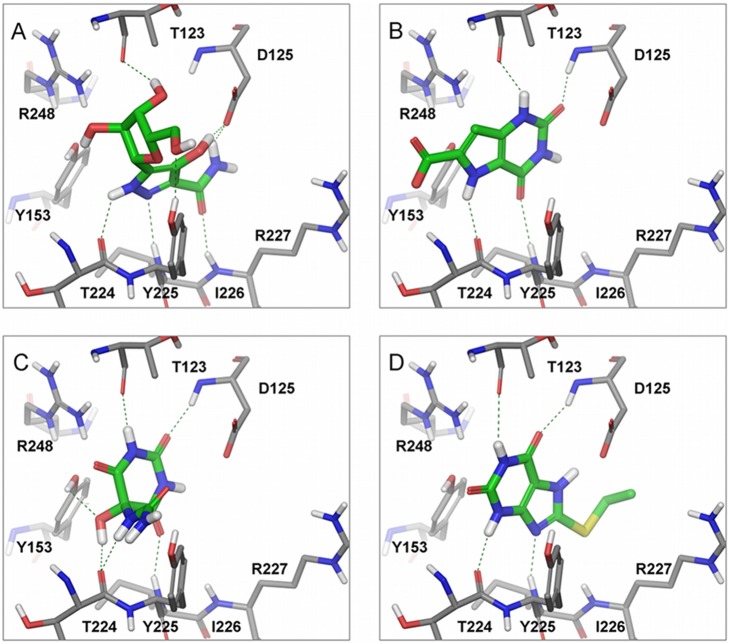
Binding modes and interaction patterns of bioactive compounds on human dyskerin. The inhibitors are located in the pseudouridylation catalytic site of the human dyskerin homology model presented in Figure 1. The binding modes are the result of an Induced Fit docking protocol for each one of the four active compounds: (*A*) compound **1**; (*B*) compound **5**; (*C*) compound **6**; (*D*) compound **10**. Hydrogen bonds are highlighted in dotted green lines. Color scheme: white, hydrogen atoms; blue, nitrogen atoms; red, oxygen atoms; yellow, sulfur atoms; gray, dyskerin model carbon atoms; green, ligand carbon atoms. The Figure was produced with Maestro software.

A more refined molecular docking technique was carried out to keep into account possible conformational changes in the active site residues, and to obtain a more accurate prediction of their binding modes. To this purpose an Induced Fit Docking (IFD) protocol was used with the human dyskerin model and the four active compounds. The IFD allows the movement of the residues in the binding site region leading to a more accurate prediction of binding modes. Compound **1** is structurally different from compounds **5**, **6** and **10** (Table 1) and the IFD results demonstrate its ability to bind the catalytic site of dyskerin with different poses. However, due to the similarity of **1** to the uridine scaffold and the fact that, according to the X-ray structure of the box H/ACA co-crystallized with bound target-RNA, the pyrimidine moiety of uridine lies in the inner pseudouridylation catalytic site (PDB ID: 3HAX), the correct binding pose of **1** is expected to be the one shown in Figure 4A [34]. Induced fit docking of compounds **5**, **6** and **10**, unveil a similar superimposition of the uracil ring (Figure 4B–D) that performs similar interactions in the pseudouridylation site. In particular, compound **1** presents two hydrogen bond interactions with the side chain of the catalytic D125, one with the pyrazolic hydroxyl group and the other with the amidic end. Conversely, compounds **5**, **6** and **10** interact with the backbone nitrogen of D125. The interactions with the side chain of the catalytic aspartate, D125, are particularly important to disrupt the formation of a salt bridge with the R227 (Figure 4), that previous structural analysis identified as the detector of the pseudouridylation conversion and subsequent substrate release [35], [36]. For all active compounds, there are conserved hydrogen bond interactions with key backbone residues of T123, G223 and Y225 (Figure 4). Importantly, compound **1** can also interact with R248 and Y153 that, together with the different interaction pattern observed with D125, can explain the higher inhibitory activity in respect to **5**, **6** and **10** (Figure 3A). Moreover, the IFD pose of compound **3** (Figure 4C) may also elucidate the slightly higher inhibitory activity in comparison to **5** and **10,** since compound **3** is able to produce additional hydrogen bonds with Y153, G223 and Y225, by exploiting the ureic and hydroxyl groups directly linked to the uridine scaffold.

It is worth to note that all these ligand-protein interactions involve polar residues with either charged or uncharged side chains. This fact highlights the polar nature of the binding pocket (Figure S8A in File S1) and also suggests the ideal region where a ligand feature should be located in order to interact optimally with the human dyskerin catalytic site. The ligand should mainly include hydrogen bonds donor and acceptor features (Figure S8B in File S1) [35]. The identification of the interaction patterns of the pseudouridylation catalytic site may be of practical use to define specific pharmacophore features useful for design of specific small-molecule modulators of human dyskerin. In fact, the comparative analysis of the available structures of related pseudouridine-synthase (e.g. PUS1 and PUS10) showed that the catalytic site is extremely conserved at the structural level [37], [38]. Therefore, it is possible that a certain degree of inhibitory capacity of the active compound **1** might be also present towards related enzymes (Figure S9A in File S1). 28S rRNA U4393 and U4390 tested in our assays are known to be specifically modified by dyskerin through the binding of snoRNAs U68 and E3, respectively [39]. To further confirm that compound **1** effect on the *in* *vitro* assay is due to its inhibitory effect on dyskerin activity, we used a different synthetic substrate RNA depleted of the sequences for snoRNAs U68 and E3 recognition and for U4390, but conserving U4393. In this case, the results were not influenced by compound **1** (Figure S9B in File S1) indicating that the identified compound is certainly active towards dyskerin pseudouridylation activity. Nevertheless, further studies aiming at modifying the chemical structure of compound **1** are necessary to achieve target selectivity and, in this context, computational insights herein presented promise to constitute a useful tool to speed-up this development.

## Conclusions

Our results indicate that it is possible to inhibit the dyskerin catalytic activity by means of low molecular weight compounds. In particular, we identified four molecules capable of significantly inhibit dyskerin pseudouridylation activity. The inhibition of the identified compounds is heterogeneous ranging from 18 to 77%. One of these compounds is pyrazofurin that was already tested as an anticancer drug. Because clinical trials with this agent did not take into account bioactivity data on human dyskerin, we cannot exclude that this compound may be readily for use in treating specific patients and/or cancer pathologies were dyskerin is overexpressed.

Overall, the revelation that small molecule compounds can inhibit the catalytic function of dyskerin and the availability of a new fast assay to detect the dyskerin pseudouridylation activity *in* *vitro* herein described, will pave the way to a pharmacological targeting of ribosome biogenesis in cancer cells. In addition, the identification of the four chemical scaffolds described will serve as a starting point to optimize novel molecules with improved pharmacological properties and biological profiles against human dyskerin.

## Methods

### Homology modeling

A homology model was created starting from the human dyskerin protein sequence (Uniprot code: O60832), by excluding nuclear and nucleolar localization sequences, which are not related to the target pseudouridylation catalytic site [40]. The resulting primary sequence (Figure S1 in File S1) was submitted to BLAST by searching on the Protein Data Bank (PDB) for the possible existence of three-dimensional homologous proteins of the human dyskerin [41]. The best homology score was found for the Cbf5-Nop10-Gar1 complex from *saccharomyces cerevisiae* (PDB ID: 3U28), with a sequence alignment identity of the 73% [16]. The pair-wise sequence alignment was performed with ClustalW and manually inspected (Figure S1 in File S1) [42].

We created the homology model of human dyskerin using the yeast template with MODELLER software (Figure 1 and Figure S1 in File S1) [43]. The model was refined and submitted to a quality check with the Procheck suite. Subsequently, a molecular dynamics simulation was performed in order to assess the quality and the folding of the constructed human dyskerin model. To this purpose the Desmond software (v3.1.51.1) was used in constructing a SPC water simulation box with standard parameters and to run the molecular simulation for 10 ns.

### Virtual screening

With the aim to discover new small molecule inhibitors targeting the human dyskerin catalytic site, the docking software Glide (version 5.7), was used to perform a high-throughput virtual screening of the open NCI (http://dtp.cancer.gov) compound dataset of the CoCoCo databases [18]. A total of around 260.000 molecules were screened by centering a 20 Å docking grid in the D125 residue, which has been described as the amino acid responsible of the pseudouridylation function of dyskerin [19], [35], [44]. Docking results were ranked based on the Glide score and the first 500 hit compounds were visually inspected taking into account several structural and physic-chemical rules, such as the qualitative evaluation of ligand-protein interactions within the active site, probability of suggested protonation and tautomeric states, stereochemistry complexity, compound availability and chemical diversity. Based on these criteria, 14 molecules (Table 1 and Figure S3 in File S1), all listed on the top of the 200 scoring hit, were acquired from the NCI for experimental testing.

### Induced fit docking

The Induced fit docking protocol present in Schrodinger Suite 2012 is a procedure that takes into account the protein flexibility in a protein-ligand docking [45], [46]. To achieve more refined docking results this protocol includes four major steps: an initial softened-potential docking, a sampling of the protein in the binding region, and finally a redocking of the ligand into low energy induced-fit structures resulted from the residues sampling followed by a scoring that reports the docking energy (GlideScore), and the receptor strain and solvation terms (Prime energy). The Induced fit docking protocol was applied to the human dyskerin model previously described and each one of the active compounds: **1**, **5**, **6** and **10**.

### Pseudouridylation assay

#### Synthetic rRNA substrate 28S U4393/U4390 design

The synthetic rRNA substrate 28S U4393/U4390 was designed to contain a 22 nucleotides long sequence of 28S rRNA with uridines U4393/U4390 and a 20 nucleotides long sequence at the 3′ to specifically reverse-transcribe the rRNA substrate. The complete sequence is here reported: (**dGd)ATCCCGAACCTGAACCGGCCAAGCGUUCAUAGCGACGCCCUAUAGUGAGUCGUAUUA(dTdC)**.

A similar synthetic sequence containing pseudouridines in positions corresponding to uridines 4393/4390 in 28S RNA was used as a control. The RNA oligonucleotides were purchased from IDT DNA.

#### Nuclear lysate preparation

Nuclear lysate was prepared from four subconfluent T175 flasks of MCF7 cells. The cells were scraped off, washed twice with 10 ml of cold phosphate-buffered saline (PBS), and swollen for 15 min in 2 times the pellet volume of a hypoosmotic homogenization buffer (10 mM HEPES pH 7.5; 1.5 mM MgCl2; 10 mM KCl and 1 mM DTT). The cells were disrupted with 12 to 15 strokes of a tight-fitting pestle in a Dounce homogenizer. After a 10-min centrifugation at 1,000 g, the pellets (crude nuclei) were suspended in 0.5 ml of reconstitution buffer (10 mM Tris-HCl pH 7.5; 3,3 mM MgCl2; 0,25 M sucrose and 1 mM DTT). The viscous solution was sonicated 6–8 times for 15 sec followed by 15-sec cooling periods in between until a great reduction of the viscosity occurred. The nuclear lysate was frozen in aliquots and stored at −80C.

#### Pseudouridylation reaction

Pseudouridylation reaction was carried out as previously described (20) with some modifications. Particularly, the reaction was carried out in a final volume of 200 µl in order to obtain a sufficient amount of RNA to be used in the consequent passages. A 200 µl reaction contained 80 µl of MCF7 nuclear extract, 40 µl of 5X Reconstitution buffer (100 mM HEPES-KOH pH 7.5; 600 mM KCl; 10 mM MgCl_2_), 8 pg of tRNA,160 U of RNasin (Promega) and approximately 80 fmol of rRNA substrate. Each compound was diluted in DMSO (Sigma-Aldrich) and 100 µM of each of them or DMSO (in the control samples) were added to the mixture. The mixture was incubated at 30° and 4°C for 120 minutes unless differently indicated. The reaction was terminated on ice and RNA extracted with Tri- Reagent (Ambion) following the manufacturer’s protocol.

#### CMC-modification of rRNA

CMC modification was carried out as previously described with some modifications [47]. To 2 µg of RNA were added 80 µl of BEU Buffer (7 M Urea, 4 mM EDTA, 50 mM Bicine-pH 8.5, final buffer pH = 8.9−9.0) and 20 µl of 1 M CMCT (Sigma–Aldrich) in BEU buffer freshly prepared. The samples were incubated for 20 minutes at 37°C and then precipitated by adding 2 µl of Pellet Paint Co-Precipitant (Merck), 50 µl of 3 M sodium acetate pH 5.5 and 600 µl of ethanol. The pellets were washed twice with 200 µl of 70% ethanol and then resuspended in 50 µl sodium carbonate buffer, pH 10.4 (50 mM sodium carbonate and 2 mM EDTA). The samples were then incubated at 37°C for 4 h. At the end of the reaction RNA was precipitated by adding 2 µl of Pellet Paint Co-Precipitant, 6 µl of 3 M sodium acetate, and 110 µl of ethanol. The pellet was then washed with 70% ethanol, resuspended in 20 µl of water and finally quantified by a nanodrop.

#### Reverse transcription reaction

The reverse transcription reaction was carried out using a specific reverse primer targeting the 20 nucleotides long sequence in the 3′ region of the synthetic rRNA substrate. The sequence of the specific reverse target is: 5′-TAATACGACTCACTATAGGG-3′.

The reaction was made in two steps in a final 20 µl mixture. During the first step 300 ng of RNA, 1 µM specific reverse primer (IDT DNA) and 40 U of RNasin Plus RNase Inhibitor (Promega) were mixed and incubate at 50° for 5 minutes in a thermocycler to stimulate the annealing of the specific reverse primer. During the second step, M-MLV Reverse Transcriptase Reaction Buffer (Promega), 200 U of M-MLV retrotranscriptase (Promega) and 10 µM dNTPs (Promega) were added to mixture and inserted in the thermocycler. The conditions of the reverse transcription were: 5 minutes at 4°C, 60 minutes at 37°C, 15 minutes at 70°C and 4°C forever.

#### Real time PCR reaction

Real-time PCR analysis was performed in a Gene Amp 7000 Sequence Detection System (Applied Biosystems) using the Syber-green (Applied Biosystems) approach. The set of primers (IDT DNA) used were: primerF 5′-GAACCTGAACCGGCCAAG-3′ and primerR 5′-CTATAGGGCGTCGCTATGA-3′. This set of primer permits the amplification of the specific retro transcribed RNA. For each sample three replicates were analyzed. The amount of 28S U4393/U4390 uridines pseudouridylated was calculated by the fold change 2∧DCt were DCt is the difference between the Ct medium of the samples treated with each molecule and the Ct medium of the positive control (the sample without the addition of inhibitors at 30°C). Real Time PCR for human TERC mRNA expression analysis was performed as previously described [48], [49], using human U6 snRNA, as endogenous control. Primers for U6 snRNA expression analysis were the following: primerF 5′-GCT GGC TTC GGC AGC ACA TAT AC-3′, primerR 5′-TATCGAACGCTTCACGAATTTGC-3′. The final results were determined by the 2∧−ΔΔCt method.

### Cell cultures and viability assay

Human breast cancer derived cell lines MCF7 (obtained from ATCC) were cultured in a monolayer at 37°C in a humidified atmosphere containing 5% CO_2_. MCF7 were grown in RPMI 1640 (Sigma-Aldrich) supplemented with 10% of fetal bovine serum (FBS, Sigma-Aldrich)), 2 mM L-glutamine (Sigma-Aldrich)), 100 U/ml penicillin, and 100 mg/ml streptomycin (Sigma-Aldrich). Cells were treated with 100 µM of each compound or DMSO for 24, 48, 72 and 96 hour. For the dose/response curve cells were treated with 5, 10, 50 and 100 µM of compound **1** for 24, 48, 72 and 96 hours. We used the Alamar Blue assay (Invitrogen) to test the vitality of the cells after the treatment following the manufacturer’s instruction. Stable MCF7 (shDKC1), and their relevant empty vector controls were generated as described previously [22].

### Pseudouridylation assay on endogenous 28S rRNA

MCF7 cells were treated with 1 µM, 10 µM and 100 µM of compound **1**, **9** or DMSO for 24 hours. Two µg of RNA was modified with CMC as described above. The reverse transcription reaction was carried out using an oligonucleotide directed to a sequence adjacent to the same 28S RNA sequence included in the synthetic rRNA substrate used in the *in* *vitro* assay: 5′- ATTATGCTGAGTGATATCCCATCGAAGGATCAAAAAGCGA-3′. The oligonucletide contained a linker sequence in its 5′ allowing the specific amplification of the reverse transcribed templates. The reaction was made in two steps in a final 20 µl mixture. During the first step 100 ng of RNA, 0.2 µM specific reverse primer and 40 U of RNasin Plus RNase Inhibitor (Promega) were mixed and incubate at 50°C for 10 minutes to allow the annealing of the specific reverse primer. During the second step, M-MLV Reverse Transcriptase Reaction Buffer (Promega), 200 U of M-MLV retrotranscriptase (Promega) and 2 µM dNTPs (Promega) were added to mixture. Reverse transcription and Real-time PCR analysis was performed as described above, using the following PCR primers: primer F 5′-GTGTCAGAAAAGTTACCACA-3′; primer R: 5′-ATTATGCTGAGTGATATCCC-3′.

### Statistical analysis

Mann-Whitney U or Fischer’s exact tests were used, when appropriate, for the comparisons among groups. Values for p less than 0.05 were regarded as statistically significant.

### Expression profile analysis

Activity and inactivity data of compound **1** were retrieved from the PubChem database (accessed Feb, 21 2013 http://pubchem.ncbi.nlm.nih.gov; CID 285701). The number of published studies where DKC1 gene is differentially expressed compared to the gene’s overall mean expression level in the study was retrieved from the Gene Expression Atlas database (accessed Feb, 21 2013 http://www.ebi.ac.uk; DKC1 homo sapiens).

## Supporting Information

File S1 **Supporting Information.** **Methods** **S1,** Pseudouridylation assay on synthetic rRNA lacking E3/U68 binding sequences. **Figure S1,** Sequence alignment of human dyskerin and yeast cbf5 primary structures. **Figure S2,** Backbone RMSD fluctuation of the human dyskerin model presented and used for structure based virtual screening during 10 ns of molecular dynamics simulation. The higher fluctuation between 7–8.5 ns is due to the movement of the loop covering the pseudouridylation site. The protein remains folded for the complete simulation time demonstrating the good quality of the assembled human dyskerin model. **Figure S3,** Chemical structures of tested compounds for inhibition of pseudouridylation catalytic activity of human dyskerin. **Figure S4,** Real time RT-PCR assay used in the pseudouridylation assay selectively amplifies full length products (5′ TAA TAC GAC TCT CTA TAG GGC GTC GCT ATG AAC GCT TGG CCG GTT CAG GTT CGG GAT 3′), while predicted truncated products (5′ TAA TAC GAC TCT CTA TAG GGC GTC GCT 3′) originating from pseudouridylated templates are not amplified. **Figure S5,** Dose-Response curve of compound 1 on MCF7 cells. The experiment was performed treating the cells with DMSO (CTRL) or 5, 10, 50 and 100 µM of compound 1 for up to 96 hours. Alamar Blue assay was performed each 24 hours. Means and SDs from three independent experiments are represented. **Figure S6,** Effect of compound **1** on telomerase RNA component (TERC) expression. The experiment was performed treating the nuclear lysate with DMSO (CTRL) or 100 µM of compound **1** for 120 minutes in the reaction mixture. Histograms represent means and SDs from three independent experiments. The final results were determined by the 2∧−ΔΔCt method. Differences between the groups are not significant. **Figure S7,** Effect of treatment with compound **9** on MCF7 endogenous U4393/U4390 rRNA pseudouridylation. Cells were treated with 1 µM, 10 µM and 100 µM of compound **9** or DMSO for 24 hours. The pseudouridylation reaction was carried out at 30°C. Histograms represent means and SEMs from three independent experiments. Differences between the groups are not significant. **Figure S8,** (*A*) Contour maps generated with the software SiteMap (http://www.schrodinger.com), thereby presenting hydrophobic (yellow regions), donor (blue regions) and acceptor (red regions) potentials. Contour maps represent the ideal region of the space where a corresponding ligand feature should be located in order to interact optimally with the human dyskerin catalytic site. (*B*) Molecular surface of the human dyskerin coloured by electrostatic potential. Blue regions represent positively charged residues while red regions negatively charged residues. **Figure S9,** (*A*) Comparative analysis of the available structures of related pseudouridine-synthase PUS1 and PUS10. On the left column, compound **1** complexes were minimized into the active site of PUS1 and PUS10 starting from the binding pose obtained by overlaying these structures with the human Dyskerin – compound **1** complex (Yellow lines indicate hydrogen bonds); On the right column, a ligand interaction diagram for each complex is presented (Grey highlight - solvent exposed; blue arrow - side chain hydrogen bond; blue dotted arrow - backbone hydrogen bond; red ball - negative charged residue; blue ball - positive charged residue; green ball - hydrophobic residue; light blue ball - polar residue). (*B*) Specific inhibitory effect of compound **1** on dyskerin activity. A different synthetic substrate RNA depleted of the sequences for snoRNAs U68 and E3 recognition and for U4390, but conserving U4393 was used in the in vitro assay developed. The experiment was performed treating the nuclear lysate with DMSO (CTRL) or 100 µM of compound **1** for 120 minutes in the reaction mixture. Histograms represent means and SEMs from three independent experiments. Correlations between the groups are not significant. **Table S1,** List of NCI human tumor cell lines with confirmed bioactivity of compound **1** and their relative expression profiles of human dyskerin classified as overexpressed, underexpressed or not differentially expressed. Data were collected as described in the materials and methods paragraph. **Table S2,** List of NCI human tumor cell lines with confirmed inactivity of compound **1** and their relative expression profiles of human dyskerin classified as overexpressed, underexpressed or not differentially expressed. Data were collected as described in the materials and methods paragraph.
(PDF)
