# Supplementary material for: Inhibition of Human Dyskerin as a New Approach to Target Ribosome Biogenesis
Source: PLoS One. 2014 Jul 10;9(7):e101971. doi: 10.1371/journal.pone.0101971 (PMC4092089; doi:10.1371/journal.pone.0101971)
Supplement: File S1 — Supporting Information. Methods S1, Pseudouridylation assay on synthetic rRNA lacking E3/U68 binding sequences. Figure S1, Sequence alignment of human dyskerin and yeast cbf5 primary structures. Figure S2, Backbone RMSD fluctuation of the human dyskerin model presented and used for structure based virtual screening during 10 ns of molecular dynamics simulation. The higher fluctuation between 7–8.5 ns is due to the movement of the loop covering the pseudouridylation site. The protein remains folded for the complete simulation time demonstrating the good quality of the assembled human dyskerin model. Figure S3, Chemical structures of tested compounds for inhibition of pseudouridylation catalytic activity of human dyskerin. Figure S4, Real time RT-PCR assay used in the pseudouridylation assay selectively amplifies full length products (5′ TAA TAC GAC TCT CTA TAG GGC GTC GCT ATG AAC GCT TGG CCG GTT CAG GTT CGG GAT 3′), while predicted truncated products (5′ TAA TAC GAC TCT CTA TAG GGC GTC GCT 3′) originating from pseudouridylated templates are not amplified. Figure S5, Dose-Response curve of compound 1 on MCF7 cells. The experiment was performed treating the cells with DMSO (CTRL) or 5, 10, 50 and 100 µM of compound 1 for up to 96 hours. Alamar Blue assay was performed each 24 hours. Means and SDs from three independent experiments are represented. Figure S6, Effect of compound 1 on telomerase RNA component (TERC) expression. The experiment was performed treating the nuclear lysate with DMSO (CTRL) or 100 µM of compound 1 for 120 minutes in the reaction mixture. Histograms represent means and SDs from three independent experiments. The final results were determined by the 2∧−ΔΔCt method. Differences between the groups are not significant. Figure S7, Effect of treatment with compound 9 on MCF7 endogenous U4393/U4390 rRNA pseudouridylation. Cells were treated with 1 µM, 10 µM and 100 µM of compound 9 or DMSO for 24 hours. The pseudouridylation reaction was carried out at [file pone.0101971.s001.pdf]

Supplementary Information

Inhibition of human dyskerin as a new approach to target ribosome biogenesis

Laura Rocchi[1,2, #], Arménio J. M. Barbosa[1, #], Carmine Onofrillo[1,3], Alberto Del Rio[1,4*], Lorenzo Montanaro[1,*]

1 Department of Experimental, Diagnostic and Specialty Medicine, Alma Mater Studiorum, University of Bologna, Bologna, Italy

2 Surgical Pathology Unit, University Hospital of Parma, Parma, Italy

3 "Giorgio Prodi" Interdepartmental Cancer Research Centre, Alma Mater Studiorum, University of Bologna, Bologna, Italy

4 Institute for Organic Chemistry and Photoreactivity, National Research Council, Bologna, Italy

[#]L.R. and A.J.M.B. contributed equally to this work.

[*] E-mail: alberto.delrio@gmail.com or lorenzo.montanaro@unibo.it

Supplementary Methods

Pseudouridylation assay on synthetic rRNA lacking E3/U68 binding sequences

The pseudouridylation assay was performed as described for the synthetic 28S rRNA substrate U4393/U4390 with the specified modifications. The synthetic rRNA substrate 28S lacking E3/U68 binding sequence was designed to contain a 9 nucleotides long sequence of 28S rRNA, which include only the Uridine 4393, and a 20 nucleotides long sequence at the 3’ to specifically reverse-transcribe the rRNA substrate. The complete sequence is here reported: (dGd)ATCCCGAACCTGAACCGGCCAAGUCCCUAUAGUGAGUCGUAUUA(dTdC). The reverse transcription was carried out using a reverse primer with the following sequence: 5’-ATTATGCTGAGTGATATCCCTAATACGTCTCACTATAGGG-3’. Real time PCR was performed using the following primers: primerF 5’-GAACCTGAACCGGCCAAG-3’; primerR 5’-ATTATGCTGAGTGATATCCC.

Supplementary Figures

```
Human Dyskerin     EFLIKPESKVAKLDTSQWPLLLKNFDKLNVRTTHYTPLACGSNPLKREIGDYIRTGFINL 60
Yeast cbf5         DFVIKPEAAGASTDTSEWPLLLKNFDKLLVRSGHYTPIPAGSSPLKRDLKSYISSGVINL 60
                   :*:****:  *. ***:*********** **: ****:..**.****:: .** :*.***

Human Dyskerin     DKPSNPSSHEVVAWIRRILRVEKTGHSGTLDPKVTGCLIVCIERATRLVKSQQSAGKEYV 120
Yeast cbf5         DKPSNPSSHEVVAWIKRILRCEKTGHSGTLDPKVTGCLIVCIDRATRLVKSQQGAGKEYV 120
                   ***************:**** *********************:**********.******

Human Dyskerin     GIVRLHNAIEGGTQLSRALETLTGALFQRPPLIAAVKRQLRVRTIYESKMIEYDPERRLG 180
Yeast cbf5         CIVRLHDALKDEKDLGRSLENLTGALFQRPPLISAVKRQLRVRTIYESNLIEFDNKRNLG 180
                    *****:*::. .:*.*:**.************:**************::**:* :*.**

Human Dyskerin     IFWVSCEAGTYIRTLCVHLGLLLGVGGQMQELRRVRSGVMSEKDHMVTMHDVLDAQWLYD 240
Yeast cbf5         VFWASCEAGTYMRTLCVHLGMLLGVGGHMQELRRVRSGALSENDNMVTLHDVMDAQWVYD 240
                   :**.*******:********:******:********** .:**:*:***:***:****:**

Human Dyskerin     NHKDESYLRRVVYPLEKLLTSHKRLVMKDSAVNAICYGAKIMLPGVLRYEDGIEVNQEIV 300
Yeast cbf5         NTRDESYLRSIIQPLETLLVGYKRIVVKDSAVNAVCYGAKLMIPGLLRYEEGIELYDEIV 300
                   * :****** :: ***.**..:**:*:*******:*****:*:**:****:***: :***

Human Dyskerin     VITTKGEAICMAIALMTTAVISTCDHGIVAKIKRVIMERDTYPRKW 346
Yeast cbf5         LITTKGEAIAVAIAQMSTVDLASCDHGVVASVKRCIMERDLYPRRW 346
                   :********.:*** *:*. :::****:**.:** ***** ***:*
```

Figure S1. Sequence alignment of human dyskerin and yeast cbf5 primary structures.

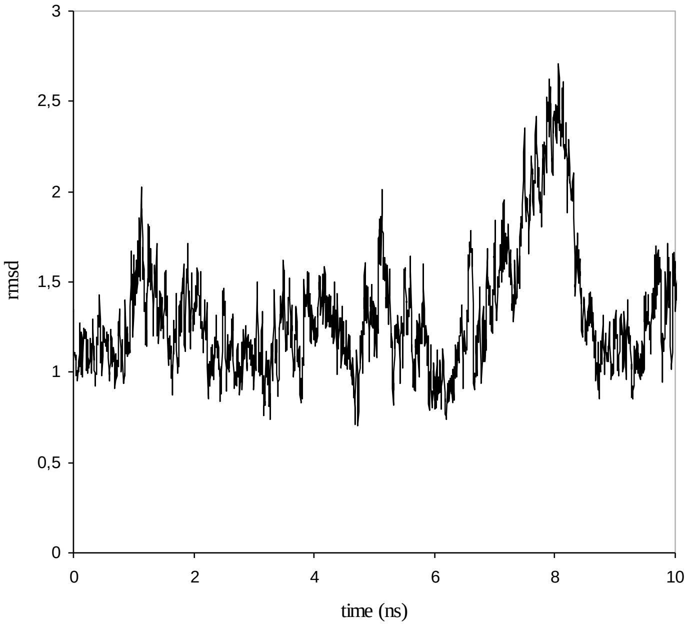

Figure S2. Backbone RMSD fluctuation of the human dyskerin model presented and used for structure based virtual screening during 10ns of molecular dynamics simulation. The higher fluctuation between 7-8.5 ns is due to the movement of the loop covering the pseudouridylation site. The protein remains folded for the complete simulation time demonstrating the good quality of the assembled human dyskerin model.

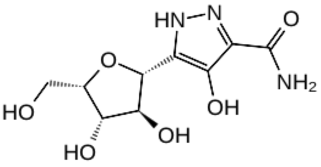 Compound **1** - NSC143095	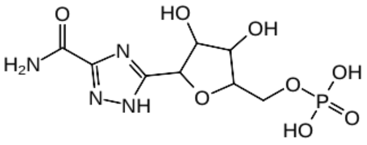 Compound **2** - NSC344552
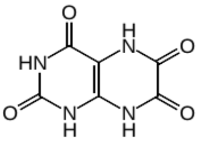 Compound **3** - NSC24507	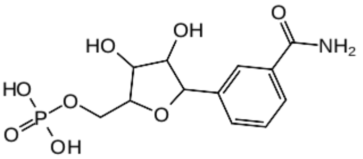 Compound **4** - NSC645648
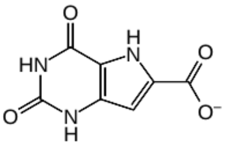 Compound **5** - NSC90991	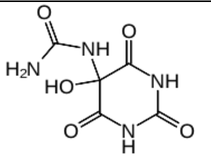 Compound **6** - NSC69824
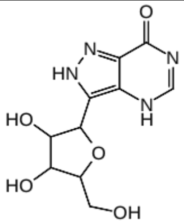 Compound **7** - NSC106486	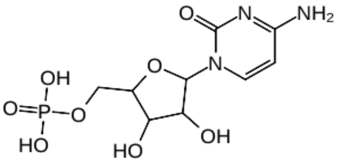 Compound **8** - NSC99445
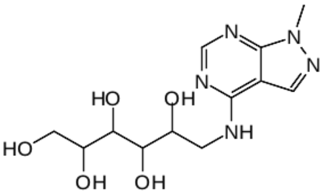 Compound **9** - NSC34520	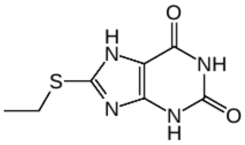 Compound **10** - NSC81499
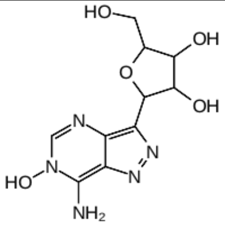 Compound **11** - NSC128669	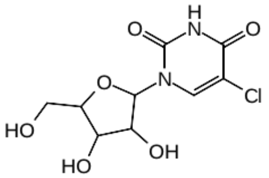 Compound **12** - NSC146433
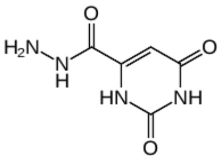 Compound **13** - NSC34715	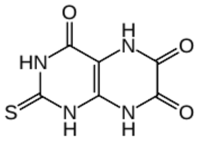 Compound **14** - NSC70443

Figure S3. Chemical structures of tested compounds for inhibition of pseudouridylation catalytic activity of human dyskerin.

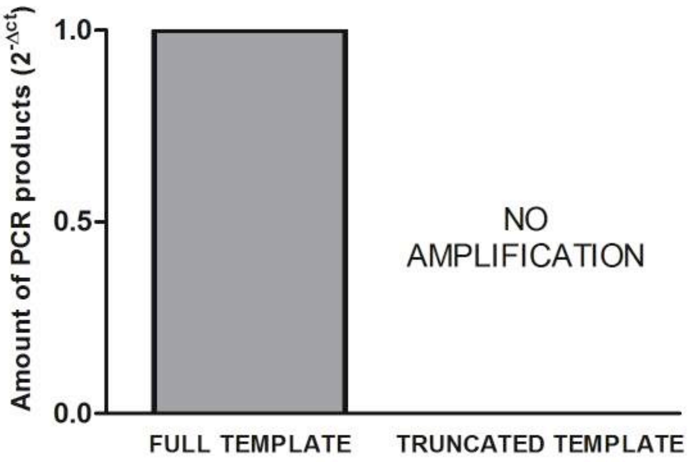

Figure S4. Real time RT-PCR assay used in the pseudouridylation assay selectively amplifies full length products (5' TAA TAC GAC TCT CTA TAG GGC GTC GCT ATG AAC GCT TGG CCG GTT CAG GTT CGG GAT 3'), while predicted truncated products (5' TAA TAC GAC TCT CTA TAG GGC GTC GCT 3') originating from pseudouridylated templates are not amplified.

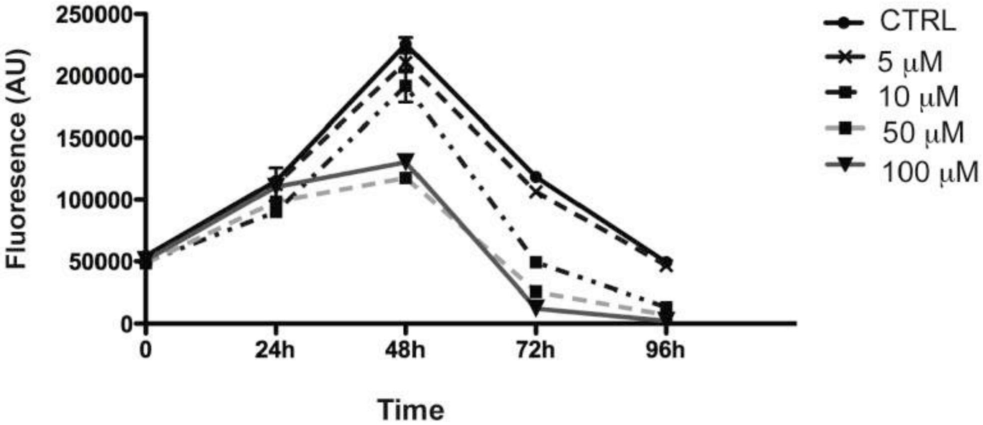

Figure S5. Dose-Response curve of compound **1** on MCF7 cells. The experiment was performed treating the cells with DMSO (CTRL) or 5, 10, 50 and 100 μM of compound 1 for up to 96 hours. Alamar Blue assay was performed each 24 hours. Means and SDs from three independent experiments are represented.

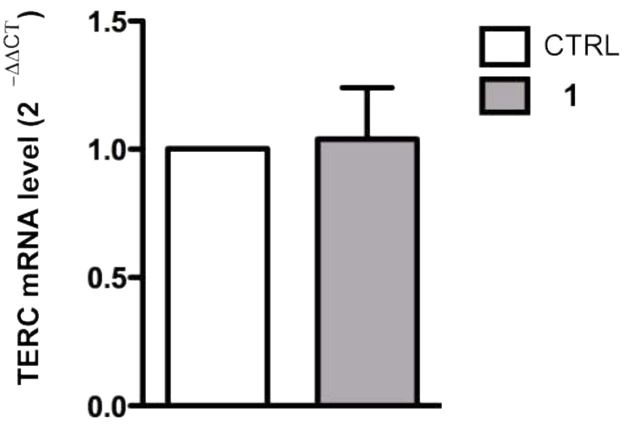

Figure S6. Effect of compound **1** on telomerase RNA component (TERC) expression. The experiment was performed treating the nuclear lysate with DMSO (CTRL) or 100 µM of compound **1** for 120 minutes in the reaction mixture. Histograms represent means and SDs from three independent experiments. The final results were determined by the 2^-ΔΔCt method. Differences between the groups are not significant.

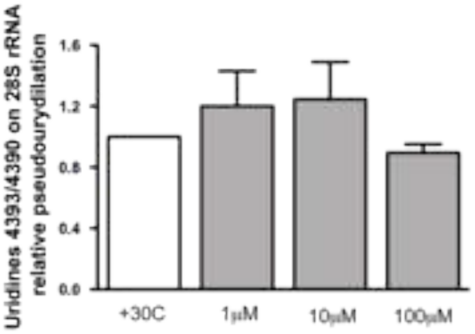

Figure S7. Effect of treatment with compound **9** on MCF7 endogenous U4393/U4390 rRNA pseudouridylation. Cells were treated with 1 µM, 10 µM and 100 µM of compound **9** or DMSO for 24 hours. The pseudouridylation reaction was carried out at 30 °C. Histograms represent means and SEMs from three independent experiments. Differences between the groups are not significant.

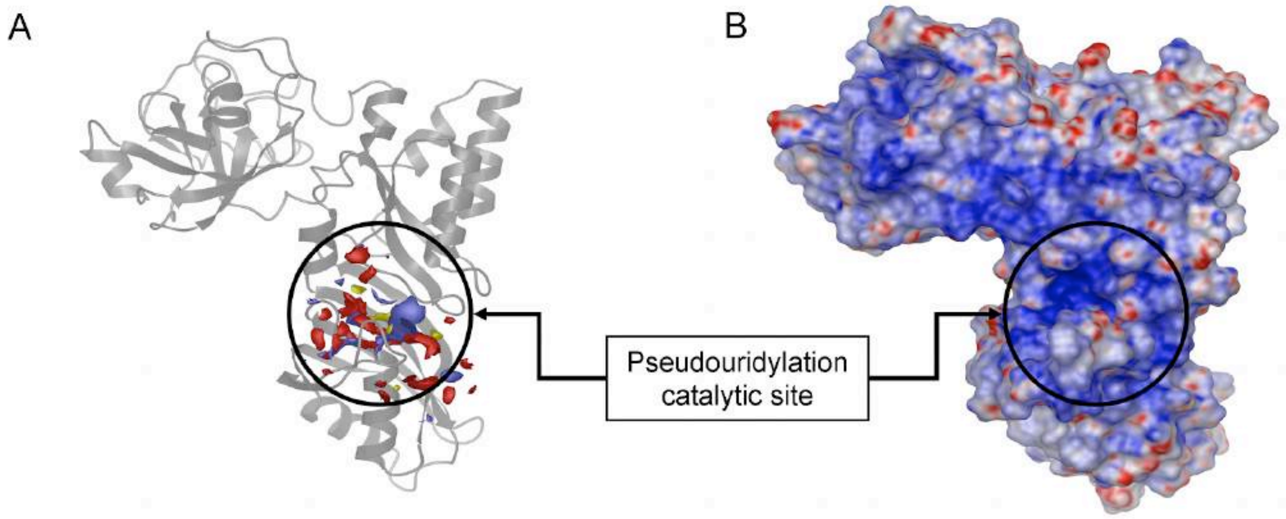

Figure S8. (*A*) Contour maps generated with the software SiteMap (http://www.schrodinger.com), thereby presenting hydrophobic (yellow regions), donor (blue regions) and acceptor (red regions) potentials. Contour maps represent the ideal region of the space where a corresponding ligand feature should be located in order to interact optimally with the human dyskerin catalytic site. (*B*) Molecular surface of the human dyskerin coloured by electrostatic potential. Blue regions represent positively charged residues while red regions negatively charged residues.

A

Dyskerin

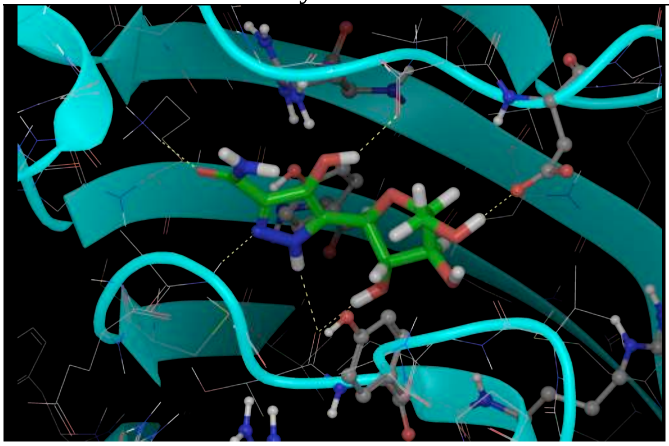

Dyskerin

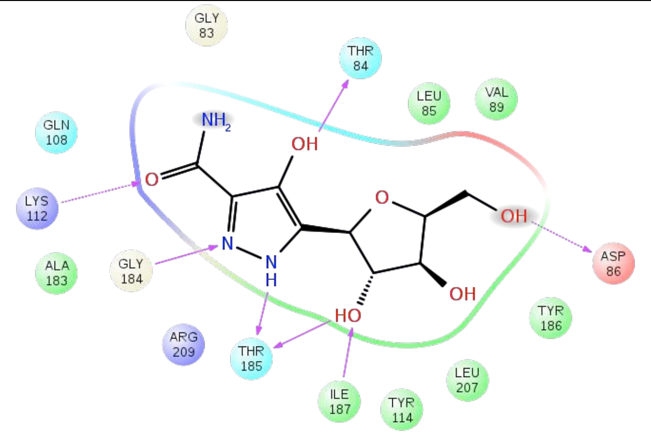

Pus1

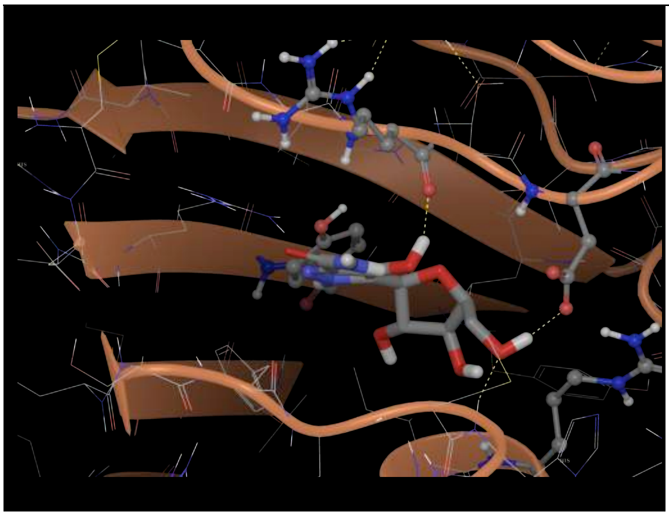

Pus1

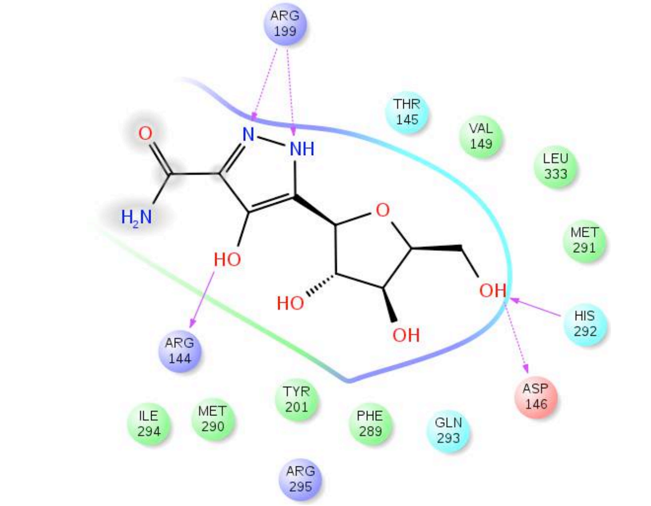

Pus10

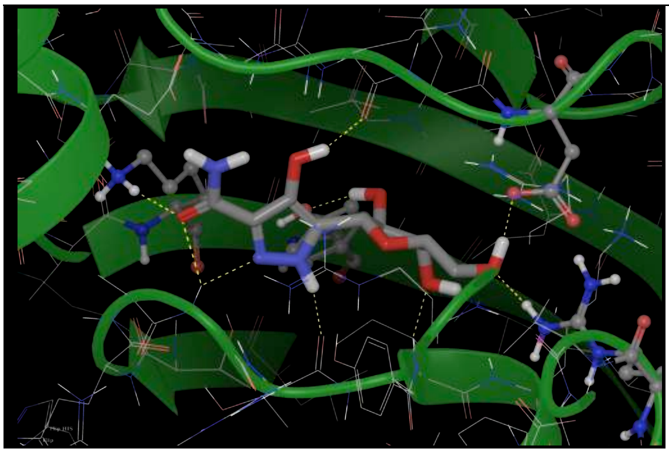

Pus10

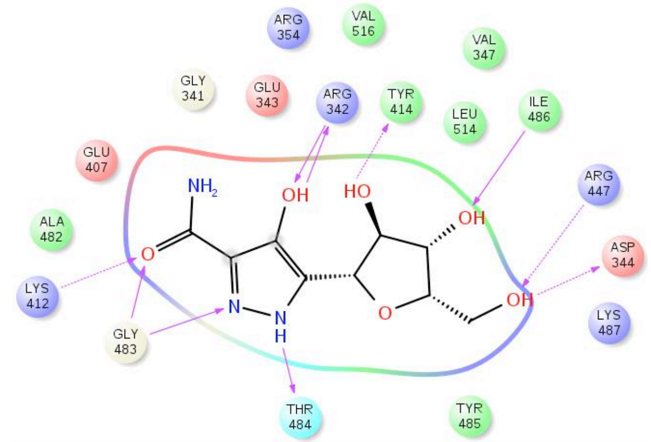

B

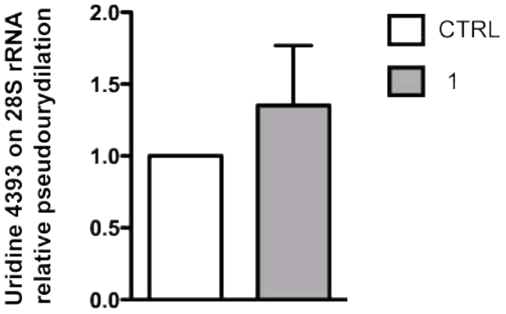

Figure S9. (*A*) Comparative analysis of the available structures of related pseudouridine-synthase PUS1 and PUS10. On the left column, compound **1** complexes were minimized into the active site of PUS1 and PUS10 starting from the binding pose obtained by overlaying these structures with the human Dyskerin – compound **1** complex (Yellow lines indicate hydrogen bonds); On the right column, a ligand interaction diagram for each complex is presented (Grey highlight - solvent exposed; blue arrow - side chain hydrogen bond; blue dotted arrow - backbone hydrogen bond; red ball - negative charged residue; blue ball - positive charged residue; green ball - hydrophobic residue; light blue ball - polar residue). (*B*) Specific inhibitory effect of compound **1** on dyskerin activity. A different synthetic substrate RNA depleted of the sequences for snoRNAs U68 and E3 recognition and for U4390, but conserving U4393 was used in the in vitro assay developed. The experiment was performed treating the nuclear lysate with DMSO (CTRL) or 100 μM of compound **1** for 120 minutes in the reaction mixture. Histograms represent means and SEMs from three independent experiments. Correlations between the groups are not significant.

Supplementary Tables

Table S1. List of NCI human tumor cell lines with confirmed bioactivity of compound **1** and their relative expression profiles of human dyskerin classified as overexpressed, underexpressed or not differentially expressed. Data were collected as described in the materials and methods paragraph.

NCI cell lines	Number of published studies		
	Overexpressed	Underexpressed	Not differentially expressed
NCI-H460 Non-Small Cell Lung	1	-	1
A549/ATCC Non-Small Cell Lung	1	1	2
LOX IMVI Melanoma	-	-	-
DU-145 Prostate	-	-	-
SF-295 Central Nervous System	-	-	-
DMS 273 Small Cell Lung	1	-	-
HT29 Colon	2	-	-
HCT-15 Colon	-	1	-
HCT-116 Colon	-	2	-
SW-620 Colon	2	-	1
IGROV1 Ovarian	-	-	-
OVCAR-8 Ovarian	-	-	-
P388 Leukemia	-	-	-
RPMI-8226 Leukemia	1	-	-
SR Leukemia	1	-	1
P388/ADR Leukemia	-	-	-
CCRF-CEM Leukemia	2	-	2
K-562 Leukemia	2	-	2
MOLT-4 Leukemia	1	-	2
HL-60(TB) Leukemia	2	-	2
SN12K1 Renal	-	-	-
CAKI-1 Renal	-	1	2
786-0 Renal	1	-	-
ACHN Renal	-	1	1

Table S2. List of NCI human tumor cell lines with confirmed inactivity of compound **1** and their relative expression profiles of human dyskerin classified as overexpressed, underexpressed or not differentially expressed. Data were collected as described in the materials and methods paragraph.

NCI cell lines	Number of published studies		
	Overexpressed	Underexpressed	Not differentially expressed
NCI-H23 Non-Small Cell Lung	1	-	-
NCI-H226 Non-Small Cell Lung	-	-	-
NCI-H322M Non-Small Cell Lung	-	1	1
HOP-62 Non-Small Cell Lung	-	-	-
HOP-18 Non-Small Cell Lung	-	-	-

HOP-92 Non-Small Cell Lung	-	-	-
NCI-H522 Non-Small Cell Lung	1	-	-
EKVX Non-Small Cell Lung	-	-	-
M14 Melanoma	-	-	-
M19-MEL Melanoma	-	-	-
MALME-3M Melanoma	-	1	-
UACC-62 Melanoma	-	-	-
UACC-257 Melanoma	-	-	-
SK-MEL-2 Melanoma	-	-	-
SK-MEL-5 Melanoma	-	1	1
SK-MEL-28 Melanoma	-	-	-
PC-3 Prostate	-	1	2
SF-268 Central Nervous System	-	-	-
SF-539 Central Nervous System	-	-	-
XF 498 Central Nervous System	-	-	-
SNB-19 Central Nervous System	-	1	1
SNB-75 Central Nervous System	-	-	-
SNB-78 Central Nervous System	-	-	-
U251 Central Nervous System	-	-	-
DMS 114 Small Cell Lung	-	1	-
COLO 205 Colon	-	-	-
DLD-1 Colon	-	-	-
KM12 Colon	-	-	-
KM20L2 Colon	-	-	-
HCC-2998 Colon	-	-	-
MCF7 Breast	3	2	3
MDA-MB-435 Breast	1	-	-
MDA-N Breast	-	-	-
BT-549 Breast	-	-	-
T-47D Breast	-	2	1
NCI/ADR-RES Breast	-	-	-
MDA-MB-231/ATCC Breast	-	1	1
HS 578T Breast	-	1	-
OVCAR-3 Ovarian	-	-	-
SK-OV-3 Ovarian	2	1	1
OVCAR-4 Ovarian			-
OVCAR-5 Ovarian			-
A498 Renal		1	2
RXF 393 Renal			-
TK-10 Renal			-
UO-31 Renal			-
SN12C Renal			-
